# International students’ study-related burnout: Associations with perceptions of the teaching-learning environment and approaches to learning

**DOI:** 10.3389/fpsyg.2022.941024

**Published:** 2022-10-13

**Authors:** Yufan Yin, Auli Toom, Anna Parpala

**Affiliations:** Centre for University Teaching and Learning, Faculty of Educational Sciences, University of Helsinki, Helsinki, Finland

**Keywords:** study-related burnout, teaching-learning environment, approaches to learning, higher education, international students

## Abstract

International students study in new, unfamiliar teaching-learning environments (TLEs) and may thus experience study-related burnout. However, little research exists on the relationship between perceptions of the TLE and such burnout, especially among international students. Nevertheless, one key factor is thought to be students’ approaches to learning. This study investigated the relationship between international students’ perceptions of the TLE, approaches to learning and study-related burnout and how these approaches mediate the relationship between perceptions of the TLE and burnout. The data were collected among international students (n = 162) in a research-intensive Scandinavian university and analyzed using confirmatory factor analyses and structural equation modelling. The results indicated that international students’ study-related burnout correlated negatively with perceptions of the TLE (alignment, interest and relevance, constructive feedback and peer support). Their study-related burnout was positively related to the unreflective approach to learning and negatively related to the deep approach to learning and organized studying. The study proved that approaches to learning acted as mediators between perceptions of the TLE and study-related burnout. The findings indicated that how the dimensions of study-related burnout were affected by different constructs of perceptions of the TLE and approaches to learning among international students. Based on these findings, the study provides implications for improving teaching. Future research should focus on the relationship between the deep approach to learning and exhaustion and how peer support affects study-related burnout.

## Introduction

International students come to study in a new, unfamiliar teaching-learning environment (TLE) (Jin and Schneider, 2019; Smith et al., 2022). Though this environment might superficially resemble that of their domestic institution due to mobility programs, credit transfer and the internationalization of curriculums (Beerkens et al., 2016), there can still be differences in how teaching and learning are organized (Lin and Scherz, 2014; Jin and Schneider, 2019). Consequently, international students must learn how to learn in the new TLE. While international students possess their own study experience from their home countries and have developed strategies that may be successful or suitable in that context (Sakurai et al., 2016; Sakurai, 2021; Smith et al., 2022), in the host institutions, they must adjust their learning processes and attempt to adapt them to the requirements and characteristics of the new TLE (Lin and Scherz, 2014; Tian et al., 2021).

International students may experience burnout in their studies (Jin et al., 2021). International students’ stress has found to be related to their experiences of the TLE (Sakurai et al., 2016; Mitchell et al., 2017) and burnout (Lin and Huang, 2014). Though research focuses on international students’ perceptions of the TLE (Sakurai et al., 2016) and well-being (Jin et al., 2021), the relationship between perceptions of the TLE and study-related burnout has not been examined in these studies among international students, especially in Finnish context. Perceptions of the TLE have found to be related to study-related burnout among non-international students (Dyrbye et al., 2009; Meriläinen, 2014; Asikainen et al., 2022). Nevertheless, the constructs of perceptions of the TLE have been developed in recent years.

**Figure 1:**
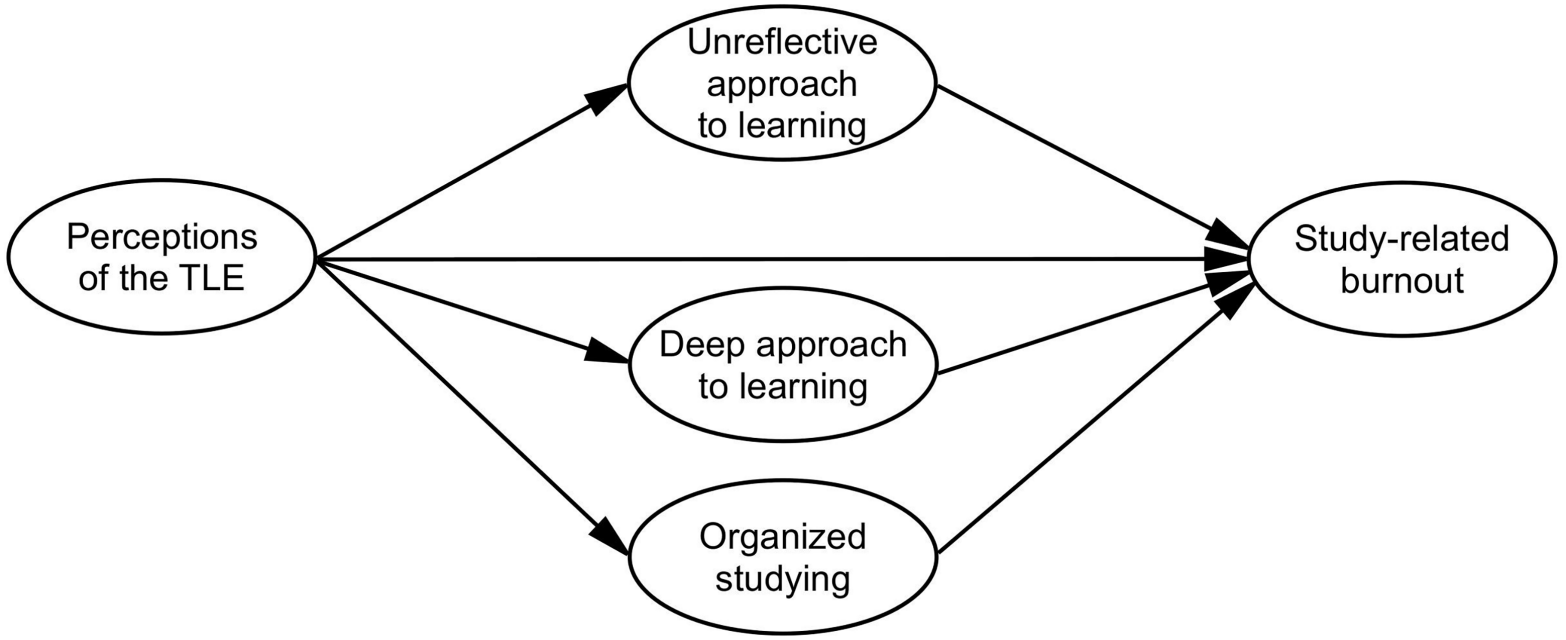
Hypothetical model of the interrelations between perceptions of the TLE, approaches to learning and perceived study-related burnout.

Furthermore, few research has examined what mediates the relationship between perceptions of the TLE and study-related burnout (Meriläinen, 2014). Student’s approaches to learning have been shown to be related both to perceptions of the TLE (Asikainen et al., 2014; Postareff et al., 2018) and to study-related burnout (Asikainen et al., 2019). Approaches to learning have also been shown to act as mediators between the TLE and learning outcomes (Lizzio et al., 2002; Rytkönen et al., 2012). Thus, the present study aims to explore the relationship between international students’ perceptions of the TLE, approaches to learning and study-related burnout and to test how approaches to learning could act as mediators between perceptions of the TLE and such burnout. The research questions are as follows (see the hypothetical model in Figure 1):

1. How are perceptions of the TLE related to approaches to learning and study-related burnout among international students?
2. How are approaches to learning related to study-related burnout among international students?
3. How do approaches to learning mediate the relationship between perceptions of the TLE and study-related burnout?

### Students’ study-related burnout

Study-related burnout in higher education, or academic burnout, has been distinguished from general burnout (Maslach et al., 1996; Asikainen et al., 2022). It has been explored with its own scales which have been adapted among students (Schaufeli et al., 2002; Hernesniemi et al., 2017). Study-related burnout refers to the psychological syndrome consisting of study-related exhaustion, cynicism and a sense of inadequacy (Schaufeli et al., 2002; Salmela-Aro et al., 2009; Salmela-Aro and Read, 2017). Study-related exhaustion involves chronic fatigue, tiredness and a lack of emotional energy (Salmela-Aro et al., 2009). Cynicism, in turn, is defined as a loss of interest in and indifference to studying and a sense of its meaninglessness (Salmela-Aro et al., 2009). Finally, a sense of inadequacy, also termed inefficacy, refers to ‘diminished feelings of competence’, and a lack of a sense of accomplishment and achievement (Salmela-Aro et al., 2009, p. 48). Study-related burnout develops when students feel that they fail to meet the study requirements and are unable to adapt themselves to those requirements despite their best efforts (Salmela-Aro and Read, 2017; Asikainen et al., 2022). Nevertheless, little research exists on how international students experience study-related burnout and how such burnout is affected by their adaptation to the new context.

### Students’ perceptions of the teaching-learning environment

According to theory and recent research among non-international students in different contexts, perceptions of the teaching-learning environment have been classified as the following constructs: alignment, interest and relevance, constructive feedback and peer support (Parpala and Lindblom-Ylänne, 2012; Asikainen et al., 2014). Alignment emphasizes that the aims of teaching are constructively in line with the content, process and assessment methods (Entwistle et al., 2002; Hailikari et al., 2021). Interest and relevance, also termed relevance and evoking interest, focuses on students’ interests in the learning content and participation in the courses, and how students perceive the relevance of the learning content (Entwistle et al., 2002; Parpala and Lindblom-Ylänne, 2012). Constructive feedback emphasizes the sufficiency of the feedback provided on students’ work and the benefits that such feedback produces, such as allowing students to form connections with their existing knowledge, removing ambiguity and improving their ways of learning and studying (Entwistle et al., 2002; Parpala and Lindblom-Ylänne, 2012). Peer support, also referred to as support from other students, focuses on the accessibility, usefulness and emotional characteristics of such assistance (Entwistle et al., 2002; Parpala and Lindblom-Ylänne, 2012).

International undergraduate students generally have positive perceptions of feedback both before and after assessment submission (Henderson et al., 2021). When dealing with academic challenges, international students ask teachers for help and value their feedback (Lin and Scherz, 2014). Moreover, peer support helps international students achieve better engagement (Mitchell et al., 2017) and it affects well-being and burnout particularly among non-international students (Lin and Huang, 2012; Räisänen et al., 2020).

### Students’ approaches to learning

Approaches to learning concern students’ intentions and learning processes during their studies (Entwistle and Ramsden, 1983; Gijbels et al., 2005). Research has identified three approaches to learning among non-international university students: the surface approach to learning, the deep approach to learning, and organized studying (Marton and Säljö, 1976; Entwistle, 2009; Lindblom-Ylänne et al., 2019; Parpala et al., 2021a). The same three approaches to learning have been found among international students in Finland (Sakurai et al., 2014, 2016). Recent research highlights that the surface approach to learning should be labelled an unreflective approach to learning, as it is founded on a fragmented knowledge base and a lack of reflection (Lindblom-Ylänne et al., 2019). The deep approach to learning, by contrast, entails using evidence and integrating new information with previous knowledge (Entwistle, 2009). Organized studying, in turn, refers to students’ ability to time and effort management (Entwistle, 2009).

### Relationship between students’ perceptions of the teaching-learning environment, study-related burnout and approaches to learning

The relationships between students’ perceptions of the TLE, their approaches to learning and study-related burnout have not been examined among international students. Moreover, while studies among non-international students have focused on these relationships separately (Tackett et al., 2017; Postareff et al., 2018; Asikainen et al., 2019; Henderson et al., 2021), there is lack of research that takes all these aspects into account. Among non-international students, the unreflective approach to learning has proven to be negatively related to perceptions of the TLE (Herrmann et al., 2017). The deep approach to learning and organized studying have proven to be positively related to perceptions the TLE among non-international students (Lizzio et al., 2002; Herrmann et al., 2017) and international students (Sakurai et al., 2016). Some constructs of perceptions of the TLE, e.g., interest and relevance, have proven to be negatively related to study-related burnout among non-international students (Asikainen et al., 2022). Moreover, a decrease in students’ satisfaction with constructive feedback and the support provided by peers explains an increased risk of study-related burnout (Dyrbye et al., 2009). Approaches to learning have also proven to be associated with study-related burnout among non-international students (Asikainen et al., 2019). For example, the surface approach to learning has been found to be positively associated with burnout (McManus et al., 2004; Asikainen et al., 2019; Hu and Yeo, 2020). Conversely, the deep approach to learning and organized studying have shown to be negatively related to burnout (McManus et al., 2004; Asikainen et al., 2019; Hu and Yeo, 2020).

Research has found that some factors (e.g., achievement motivation and proper workload) act as mediators between perceptions of the TLE and study-related burnout (Meriläinen, 2014). Furthermore, approaches to learning have proven to act as mediators between perceptions of the TLE and learning outcomes such as examination grades (Lizzio et al., 2002; Diseth et al., 2006, 2010; Rytkönen et al., 2012). In light of these previous studies, when exploring the relationship between perceptions of the TLE and study-related burnout, the mediating effects of approaches to learning should be taken into account.

## Materials and methods

### Research context

In 2018, 12% of first-time graduates at master’s or equivalent levels in Finland were international students (*cf.* an average of 19% in OECD countries; OECD, 2020). At the research-intensive Finnish university where the study was conducted, the total proportion of international students was 14.5%. In 2018, 1,150 bachelor and master’s students whose nationality was not Finnish were registered on the Student Register database. At that time, this university offered 37 bachelor (180 ECTS, 3 years) and 65 master’s programs (120 ECTS, 2 years). The Faculties of Agriculture and Forestry, Arts, Educational Sciences, Biosciences, Science, and Social Sciences offered more programs than did the Faculties of Law, Pharmacy, Theology, and Medicine. Before the first bachelor program was taught fully in English in autumn 2019, all bachelor programs had been taught in Finnish or Swedish. By contrast, the main language of instruction of master’s programs had been English (90.1% in the present study).

### Participants

A total of 162 international students from 10 faculties participated in the study in 2018 and 2019. Students were informed about the purpose of the study and participated voluntarily. The distribution of participants was in line with that of registered international students in the university; more information about gender, student status, and faculties is displayed in Table 1. The age of the participants ranged from 19 to 47 years (M = 26, SD = 5.2), and more than two thirds of them were female. The participants came from 46 countries and thus represented diverse cultural and educational backgrounds.

**Table 1:** Participant demographics.

		No. of students	%	University register office (%)
Gender	Female	113	69.8	59.4
	Male	48	29.6	40.6
	Unknown	1	0.6	
Student status	Degree-seeking Bachelor	8	4.9	20.3
	Degree-seeking Master	78	48.1	57.6
	Visiting Bachelor	36	22.2	22.1
	Visiting Master	40	24.7	
Faculty	Arts, humanities, and social sciences	89	54.9	51.3
	Sciences	73	45.1	48.7
Year of study	1st	83	51.1	
	2nd	38	23.5	
	3rd	19	11.7	
	4th or more	16	9.9	
	unknown	6	3.7	
Country of origin	Europe	89	54.9	
	Asia	31	19.1	
	North America	17	10.5	
	Africa	5	3.1	
	South America	4	2.5	
	Oceania	3	1.9	
	Dual nationality	5	3.1	
	Unknown	8	4.9	

### Measures

The data concerning approaches to learning and experiences of the TLE were collected using the HowULearn Questionnaire (in English). The HowULearn questionnaire (Parpala and Lindblom-Ylänne, 2012) has been validated in Finnish and other cultural contexts (Rytkönen et al., 2012; Cheung et al., 2020; Parpala et al., 2021b). Students responded to all items on a five-point scale from 1 (completely disagree) to 5 (fully agree).

Perceptions of the TLE were measured by 14 items originating from the Experiences of Teaching and Learning Questionnaire (ETLQ; Entwistle et al., 2003; Parpala et al., 2013). The following four dimensions have proven to be robust in previous research (Herrmann et al., 2017) and were used in the present study: alignment (4 items), interest and relevance (3 items), constructive feedback (3 items), and peer support (4 items).

On the items measuring approaches to learning, students described their study practices regarding their programs as a whole (Entwistle et al., 2002; Parpala and Lindblom-Ylänne, 2012). The subscales consisted of the unreflective approach to learning (4 items), the deep approach to learning (4 items) and organized studying (4 items; Parpala and Lindblom-Ylänne, 2012).

Study-related burnout was measured by a modified version of the School Burnout Inventory (SBI; Salmela-Aro et al., 2009), which has been validated in the Finnish context (Räisänen et al., 2020). These items were classified as exhaustion (3 items), cynicism (4 items) and a sense of inadequacy (2 items) (Salmela-Aro et al., 2009).

The last section, regarding background information, included gender, age, faculty, student status and length of study.

### Data analyses

Confirmatory factor analysis was performed using SPSS 27 to examine the factors related to perceptions of the TLE, approaches to learning, and study-related burnout. One item on the unreflective approach to learning (‘Often I have to repeat things in order to learn them’) was deleted. This was because the estimate of standardized regression weights was low (0.13), and removing the item contributed to an improvement of.10 in Cronbach’s α. Reliability coefficient alphas were all sufficient, ranging between.66 and.88 (see Table 2). The results of the approaches to learning and study-related burnout exhibited a reasonable fit (*df* = 41, *χ*^2^ = 61.726, *p* = 0.020, GFI = 0.937, CFI = 0.962, TLI = 0.949, RMSEA = 0.056, SRMR = 0.058 and *df* = 24, *χ*^2^ = 46.777, *p* = 0.004, GFI = 0.860, CFI = 0.967, TLI = 0.951, RMSEA = 0.077, SRMR = 0.056 respectively). By contrast, the results of perceptions of the TLE exhibited a poor model fit (*df* = 71, *χ*^2^ = 223.741, *p* < 0.001, GFI = 0.846, CFI = 0.847, TLI = 0.804, RMSEA = 0.116, SRMR = 0.080), which supports the choice of exploring them separately in most previous studies.

**Table 2:** Reliability, descriptive statistics, and correlations.

**Factors**	**1**	**2**	**3**	**4**	**5**	**6**	**7**	**8**	**9**	**10**
1. Alignment										
2. Interest and relevance	0.62^**^									
3. Constructive feedback	0.69^**^	0.48^**^								
4. Peer support	0.35^**^	0.45^**^	0.37^**^							
5. Unreflective approach to learning	−0.35^**^	−0.42^**^	−0.24^**^	−0.18^*^						
6. Deep approach to learning	0.32^**^	0.47^**^	0.27^**^	0.44^**^	−0.49^**^					
7. Organized studying	0.29^**^	0.27^**^	0.23^**^	0.29^**^	−0.30^**^	0.41^**^				
8. Exhaustion	−0.31^**^	−0.23^**^	−0.34^**^	−0.18^*^	0.37^**^	−0.13	−0.11			
9. Cynicism	−0.45^**^	−0.52^**^	−0.39^**^	−0.20^*^	0.45^**^	−0.29^**^	−0.31^**^	0.56^**^		
10. Sense of inadequacy	−0.24^**^	−0.27^**^	−0.28^**^	−0.17^*^	0.56^**^	−0.27^**^	−0.21^**^	0.65^**^	0.50^**^	
*M*	3.58	3.91	3.45	3.94	2.14	4.07	3.56	2.50	2.44	2.66
SD	0.76	0.74	0.85	0.78	0.79	0.59	0.85	1.08	1.12	0.97
Cronbach’s α	0.81	0.72	0.83	0.67	0.74	0.74	0.80	0.74	0.88	0.66

*p < 0.05, **p < 0.01.

Then t-test was conducted to explore the differences in gender, faculties (science or other, i.e., arts, humanities and social sciences) or length of study (1 year or more). No statistically significant differences were found. Bivariate correlation analysis was performed to examine the relationship between those variables. Structural equation modeling with maximum likelihood was employed to test the hypothetical model (see Figure 1) using SPSS AMOS 27. The fit of the model examining study-related burnout as a whole was unsatisfactory. Therefore, we report the models measuring study-related burnout separately as exhaustion, cynicism and a sense of inadequacy.

## Results

### Perceptions of the TLE in relation to approaches to learning and study-related burnout

For the first research question, the results showed that correlations between perceptions of the TLE and the approach to learning were statistically significant and also in the expected directions (see Table 2). International students’ perceptions of the TLE correlated negatively, to a statistically significant degree, with the unreflective approach to learning. In turn, they were statistically significantly and positively related to the deep approach to learning and organized studying.

Moreover, perceptions of the TLE were found to correlate negatively, to a statistically significant degree, with study-related burnout. However, the correlation was slightly weaker for the relationship between perceptions of the TLE and a sense of inadequacy than for the relationship between perceptions of the TLE and exhaustion or cynicism. In addition, peer support was less strongly related to study-related burnout than were other perceptions of the TLE.

### Approaches to learning in relation to study-related burnout

For the second research question, the results showed that international students’ unreflective approach to learning correlated positively with exhaustion, cynicism, and a sense of inadequacy (see Table 2). Conversely, the deep approach to learning displayed significant negative correlations with cynicism and a sense of inadequacy, as did organized studying.

Nevertheless, the deep approach to learning and organized studying failed to display statistically significant correlations with exhaustion. This indicates that exploring study-related burnout separately in the structural model might reveal more details about how approaches to learning mediate the relationship between perceptions of the TLE and study-related burnout.

### Approaches to learning mediating the relationship between perceptions of the TLE and study-related burnout

**Figure 2:**
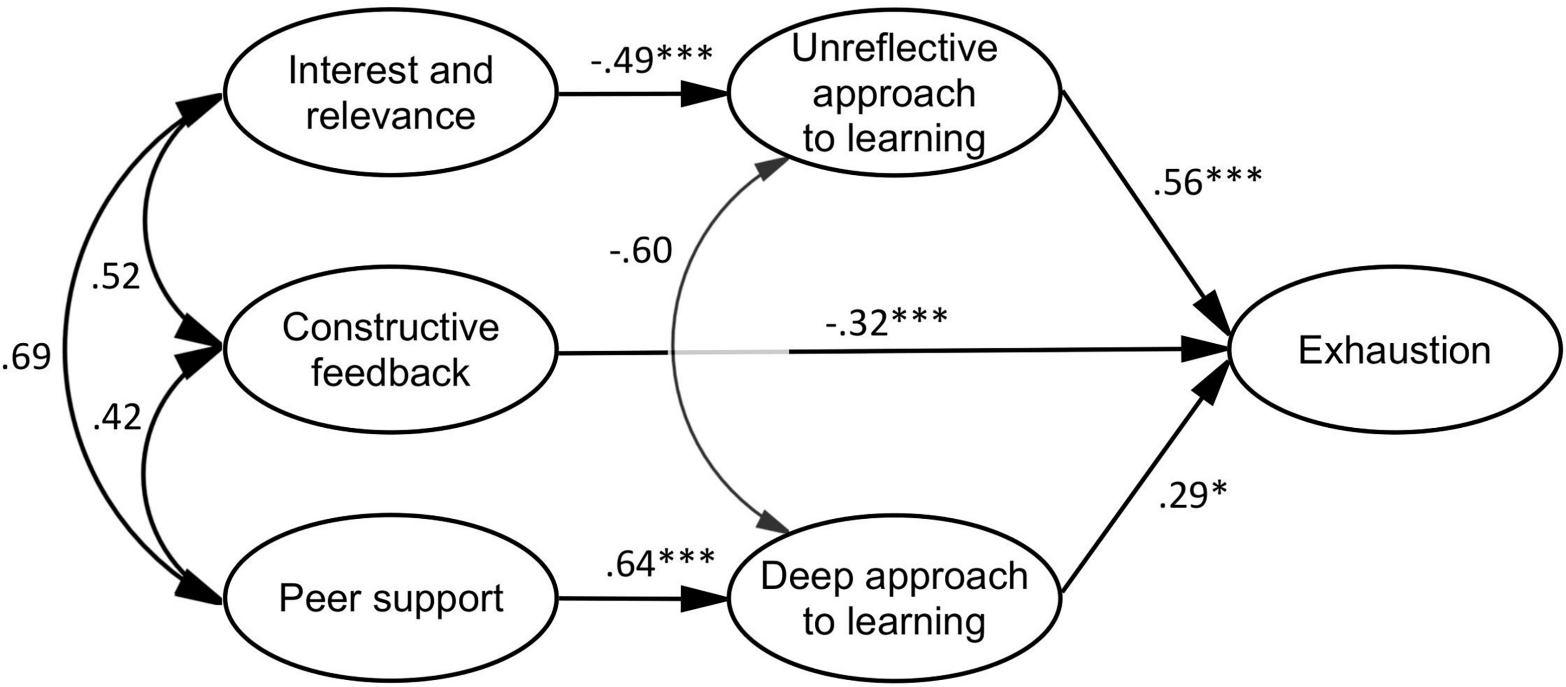
Mediation model of perceptions of the TLE, approaches to learning, and exhaustion. *p < 0.05, ***p < 0.001.

The model of perceptions of the TLE, approaches to learning, and exhaustion (*df* = 161, *χ*^2^ = 295.905, *p* < 0.001, GFI = 0.847, AGFI = 0.800, CFI = 0.881, TLI = 0.860, RMSEA = 0.072, SRMR = 0.079; see Figure 2) confirmed that interest and relevance correlated with the unreflective approach to learning negatively and statistically significantly. Moreover, a positive, statistically significant correlation was found between peer support and the deep approach to learning. The standardized direct effect of constructive feedback on exhaustion was negative and different to a statistically significantly degree. The model confirmed that the standardized direct effect of the unreflective approach to learning on exhaustion was positive and statistically significant. However, the deep approach to learning correlated positively with exhaustion, which was not in line with the hypothetical model or the bivariate correlation results. In addition, the model confirmed that the unreflective approach to learning mediated the relationship between interest and relevance and exhaustion. The standardized indirect (mediated) effect of interest and relevance on exhaustion was −0.274 (*p* < 0.001). That is, due to the mediated effect of interest and relevance on exhaustion, when interest and relevance increased by 1 standard deviation, exhaustion decreased by.274 standard deviations. Meanwhile, the mediated effect of peer support on exhaustion was positive but not statistically significant (*β* = 0.187, *p* = 0.079).

**Figure 3:**
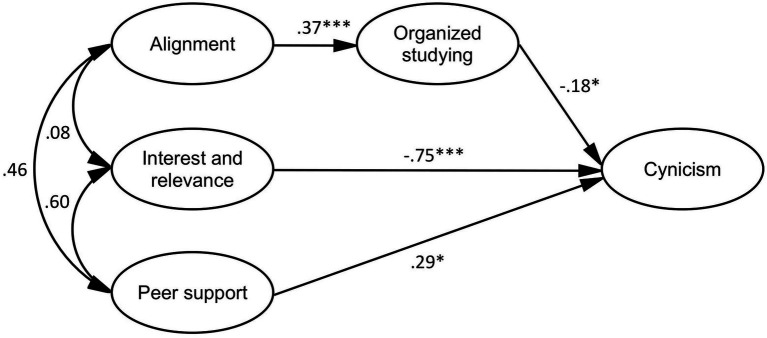
Mediation model of perceptions of the TLE, organized studying, and cynicism. *p < 0.05, ***p < 0.001.

The model of perceptions of the TLE, organized studying, and cynicism (*df* = 146, *χ*^2^ = 317.631, p < 0.001, GFI = 0.840, AGFI = 0.792, CFI = 0.869, TLI = 0.847, RMSEA = 0.085, SRMR = 0.108; see Figure 3) showed that alignment correlated positively with organized studying. The standardized direct effect of interest and relevance on cynicism was negative and statistically significant. However, the standardized direct effect of peer support on cynicism was positive, which was not in the expected direction. By contrast, the model showed that organized studying correlated negatively with cynicism. The standardized indirect effect of alignment on cynicism was negative and statistically significant (*β* = −0.067, *p* = 0.043). That is, organized studying mediated the relationship between alignment and cynicism.

**Figure 4:**
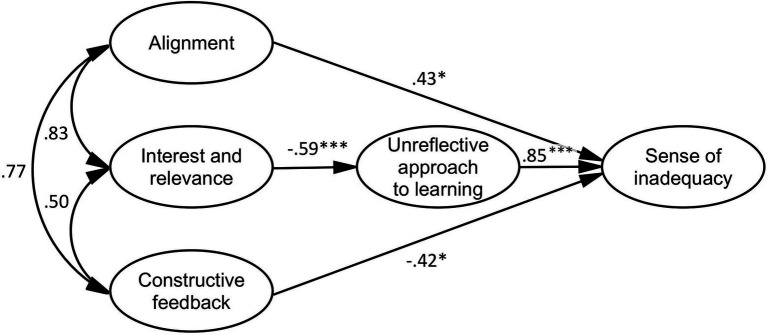
Mediation model of perceptions of the TLE, the unreflective approach to learning, and a sense of inadequacy. *p < 0.05, ***p < 0.001.

The model of perceptions of the TLE, the unreflective approach to learning, and a sense of inadequacy (*df* = 97, *χ*^2^ = 215.436, p < 0.001, GFI = 0.860, AGFI = 0.804, CFI = 0.893, TLI = 0.867, RMSEA = 0.087, SRMR = 0.072; see Figure 4) confirmed that interest and relevance correlated negatively with the unreflective approach to learning. Constructive feedback exerted a significant negative direct effect on a sense of inadequacy. By contrast, alignment had a significant positive direct effect on a sense of inadequacy. The standardized indirect effect of interest and relevance was negative and statistically significant (*β* = −0.496, p < 0.001). Therefore, the relationship between interest and relevance and a sense of inadequacy was mediated by the unreflective approach to learning.

## Discussion

Similar trends regarding learning and burnout were found among international students in the present study as in previous studies on non-international students. University students, both international and non-international students, generally perceive peer support, interest and relevance more positively than other constructs of the TLE (Asikainen et al., 2014; Herrmann et al., 2017; Cheung et al., 2020). Moreover, the majority of international and non-international university students favor the deep approach to learning and organized studying over the unreflective approach to learning (prev. Surface approach to learning; Sakurai et al., 2014; Herrmann et al., 2017). Finally, they experience a greater sense of inadequacy and exhaustion than they did cynicism about their studies (Parpala et al., 2021a; Asikainen et al., 2022).

### Relationship between students’ perceptions of the TLE, approaches to learning, and study-related burnout

The results confirmed that international students with more positive perceptions of the TLE relied less on the unreflective approach to learning and were more apt to apply the deep approach to learning and organized studying. This was in line with prior findings among non-international students (Herrmann et al., 2017). Moreover, a previous study among international students (Sakurai et al., 2016) found the same relationships between interest and relevance and approaches to learning.

The results showed that more positive perceptions of the interestingness and relevance of the TLE led to less exhaustion, cynicism and a sense of inadequacy among international students. Such negative correlations between interest and relevance and study-related burnout, especially cynicism, have also been found among non-international students in the same context (Asikainen et al., 2022). Cynicism also measures students’ interest in their studies (Entwistle et al., 2002; Parpala and Lindblom-Ylänne, 2012), which could explain its strong relationship with interest and relevance.

In previous research, more positive perceptions of the TLE (peer community, faculty relationships, quality of teaching) have found to be associated with lower levels of burnout (Meriläinen, 2014; Tackett et al., 2017). However, due to recent developments in the theory and scales of perceptions of the TLE, the relationships between alignment, constructive feedback, peer support and aspects of study-related burnout have not been investigated; thus, our results constitute novel findings. First, though international students highly valued peer support, the negative correlation between peer support and study-related burnout was weaker than other perceptions of the TLE. Similarly, non-international students perceive peer support more positively than alignment and constructive feedback, while peer support displays a weaker association with study-related stress than that of other perceptions of the TLE (Cheung et al., 2020). Peer support has been regarded as an indispensable construct of the support system among both international students (Sakurai et al., 2016; Mitchell et al., 2017) and non-international students (Tackett et al., 2017). For example, non-international students’ community of peers has been found to be associated with less emotional exhaustion (Tackett et al., 2017). By contrast, Räisänen et al. (2020) found that non-international students who frequently participated in peer learning and valued peer support exhibited a higher level of exhaustion, which can be explained by peer pressure. Second, in our study, international students with a more positive perception of alignment tended to better manage their time and effort and experience less study-related burnout, especially cynicism. This result could be explained by the qualitative finding that clear expectations and carefully selected learning materials reduced international students’ feelings of uncertainty, allowing them to easily build a sense of community and trust (Lin and Scherz, 2014). Third, the results showed a negative correlation between constructive feedback and study-related burnout. In Meriläinen (2014), non-international students’ positive perceptions of the TLE were measured by approachable teachers and pedagogical counselling, and these constructs explained the lower levels of study-related burnout.

### Relationship between students’ approaches to learning and study-related burnout

The present study clarified the relationship between international students’ approaches to learning and aspects of study-related burnout. International students’ unreflective approach to learning correlated positively with exhaustion, cynicism and sense of inadequacy, which was in line with previous research among non-international students (Asikainen et al., 2019). For such international students, listening to lectures, reading material and completing assignments becomes more challenging and time consuming (Sakurai, 2021). Therefore, they are more likely to experience fatigue, tiredness and a loss of emotional energy. Such a situation also causes them to feel overwhelmed and to lack a sense of achievement.

In line with previous research among non-international students (Asikainen et al., 2019), the results indicated that international students’ deep approach to learning and organized studying were negatively related to cynicism and a sense of inadequacy. That is, if international students struggled to integrate new information with previous knowledge or to manage their time and effort, they were more likely to challenge the meaning of study, lose interest and become indifferent.

### Students’ approaches to learning as mediators between perceptions of the TLE and study-related burnout

The results showed that the relationship between international students’ perceptions of the TLE and study-related burnout was mediated by approaches to learning. This was demonstrated, first, by the varying indirect effects of interest and relevance and peer support on exhaustion. The results indicate that if international students are highly motivated and rely less on the unreflective approach to learning, they experience less exhaustion. On the other hand, the results showed that more positive perceptions of peer support, along with the use of the deep approach to learning, are related to a higher level of exhaustion. One potential explanation is that intensive interaction with other students increases the complexity of learning processes and that students applying the deep approach to learning are more likely to investigate the subject matter thoroughly. These factors increase students’ workload and thus contribute positively to exhaustion. Nevertheless, in both the present study and Asikainen et al. (2022; non-international students), the deep approach to learning correlated negatively, but below the level of statistical significance, with exhaustion. These results fail to confirm previous findings of a statistically significant negative association between non-international students’ deep approach to learning and exhaustion and a negative direct effect of the deep approach to learning on exhaustion (Hu and Yeo, 2020).

Second, the results showed that if teaching aims are congruous with the content, process and assessment of learning, it is easier for international students to schedule their studies, potentially leading to less study-related cynicism. This supports the previous result that international students’ organized studying mediated the relationship between organization and alignment and examination grades (Sakurai et al., 2016).

Third, the results showed that the negative relationship between interest and relevance and a sense of inadequacy was mediated by the unreflective approach to learning. However, it remains unclear why alignment exerted a positive direct effect on a sense of inadequacy in the model although alignment was negatively related to a sense of inadequacy. One possible explanation is that if learning tasks and goals are too strict, the activities and materials are overly organized or structured, leaving no space for students’ aims and motivation. This could cause them to become overwhelmed by the work and lose their sense of achievement.

### Practical implications

The present study has implications for improving teaching and support for students’ well-being. In teacher training, it would be important to make teachers be aware of the relationship between TLE, learning, and well-being. So that they can support students’ well-being in their teaching. Teachers should carefully select learning materials and design assignments such that they match the aims of the course. Moreover, it is necessary to ensure that students are clear about the learning task and the relevance of the content and to evoke their interest in actively participating in class. In this way, students can avoid self-doubt and experience feelings of control and a sense of achievement (Lin and Scherz, 2014). When organizing class activities, sufficient time should be allotted for student interactions, especially when the learning tasks include comprehension or when the theme is tied to students’ study experiences.

Considering the role of approaches to learning, to help international students adapt to the new TLE, teachers could provide detailed and understandable feedback to help students integrate new information with previous knowledge (Henderson et al., 2021). It is important to create a syllabus containing detailed information about the design of the course, clear expectations for submitting assignments and explicit outcomes for course participation (Lin and Scherz, 2014; Henderson et al., 2021). Moreover, if international students are clear about grading procedures, teachers’ office hours, and expectations for making appointments, they can better manage their time and keep pace with the course schedule (Lin and Scherz, 2014). In this way, they are less likely to challenge the meaning of studying and to become indifferent to courses or even their entire study program.

### Limitations and future research

The results of the present study should be seen in the context of the following limitations. First, the research is based on students’ self-reported scales. Nevertheless, the results of confirmatory factor analysis verified that the factor structure of the observed variables was acceptable. Second, due to the small number of participants, the relationship between perceptions of the TLE and burnout with consideration of disciplinary variation was unable to carry out. Disciplines have been shown to affect approaches to learning and perceptions of the TLE among international students (Tian et al., 2021) and non-international students (Parpala et al., 2010, 2021b). For example, international students in arts, humanities and social sciences perceived teacher support more negatively than did students in life sciences and medicine (Tian et al., 2021). Though the participants were drawn from several faculties and thus constituted a representative target group, it remains unclear how the direct and indirect effects in the models change among students from different faculties or disciplines. Third, also due to the small sample size, it remains unknown how country of origin affects international students’ perceptions of the TLE, approaches to learning or study-related burnout. Previous research in the same Finnish context has shown that European students less apply the surface approach to learning and organized in their studies than Asian students do, but the differences are small (Sakurai et al., 2016). Their perceptions of the TLE are not different from each other, except for teaching for understanding (Sakurai et al., 2016). The findings suggest that culture difference within international students does not lead to variety of perception of the TLE. Therefore, considering the size of European and Asian students, the study does not examine difference in country of origin.

The findings indicate that the scales measuring both learning and the risk of burnout are robust, irrespective of students’ nationality. Therefore, the present study suggests that to understand international students’ perceptions of the TLE, approaches to learning, and study-related burnout, current theories and research based on university students in general could be used. Nevertheless, the inconsistent findings on the relationship between the deep approach to learning and exhaustion and how peer support affects study-related burnout suggest the need for further qualitative research.

The study suggests that study-related burnout should be assessed along with approaches to learning to reflect the complexity of the phenomenon. The findings imply that both the unreflective and deep approaches to learning could act as mediators between perceptions of the TLE and exhaustion. However, as the present study did not adopt a personal-oriented approach, interpreting the results requires further research. For example, one previous study demonstrated that in an online teaching situation, students scoring high on the unreflective and deep approaches to learning and organized studying experienced more exhaustion than did students representing other profiles (Parpala et al., 2021a). Thus, to obtain a clearer picture of these relationships, person-oriented methods should be used. In a further study, among the two profiles scoring relatively low on the unreflective approach and high on the deep approach to learning, students with higher scores for organized studying reported less cynicism (Parpala et al., 2021a). Consequently, rather than the unreflective and deep approach to learning, organized studying emerged as the mediator between perceptions of the TLE and cynicism.

## Conclusion

This study explored the relationship between international students’ perceptions of the TLE and study-related burnout, mediated by their approaches to learning. International students’ sense of inadequacy was higher than their sense of exhaustion and cynicism. Moreover, their perceptions of peer support and interest and relevance were more positive than their perceptions of alignment and constructive feedback. Interest and relevance acted as the active construct of perceptions of the TLE affecting approaches to learning and study-related burnout. By contrast, strong positive evaluations of peer support failed to exert a strong effect on learning process or study-related burnout. On the whole, international students’ exhaustion, cynicism and sense of inadequacy were affected by different constructs of perceptions of the TLE and approaches to learning. The study proves that approaches to learning act as mediators between perceptions of the TLE and study-related burnout.

## Data availability statement

The raw data supporting the conclusions of this article will be made available by the authors, without undue reservation.

## Ethics statement

Ethical review and approval was not required for the study on human participants in accordance with the local legislation and institutional requirements. The patients/participants provided their written informed consent to participate in this study.

## Author contributions

AT, AP, and YY developed the theory, designed the work, and drafted the work and revised it. YY collected the data, and performed the analyses, and coordinated the project. All authors contributed to the article and approved the submitted version.

## Funding

This work was supported by the Chinese Scholarship Council (CSC; grant number: 201706620072) and open access was funded by Helsinki University Library.

## Conflict of interest

The authors declare that the research was conducted in the absence of any commercial or financial relationships that could be construed as a potential conflict of interest.

## Publisher’s note

All claims expressed in this article are solely those of the authors and do not necessarily represent those of their affiliated organizations, or those of the publisher, the editors and the reviewers. Any product that may be evaluated in this article, or claim that may be made by its manufacturer, is not guaranteed or endorsed by the publisher.

## References

[ref1] Asikainen H. Nieminen J. H. Häsä J. Katajavuori N. (2022). University students’ interest and burnout profiles and their relation to approaches to learning and achievement. Learn. Individ. Differ. 93:102105. doi: 10.1016/j.lindif.2021.102105

[ref2] Asikainen H. Parpala A. Lindblom-Ylänne S. Vanthournout G. Coertjens L. (2014). The development of approaches to learning and perceptions of the teaching-learning environment during bachelor level studies and their relation to study success. High. Educ. Stud. 4, 24–36. doi: 10.5539/hes.v4n4p24

[ref3] Asikainen H. Salmela-Aro K. Parpala A. Katajavuori N. (2019). Learning profiles and their relation to study-related burnout and academic achievement among university students. Learn. Individ. Differ. 78:101781. doi: 10.1016/j.lindif.2019.101781

[ref4] Beerkens M. Souto-Otero M. de Wit H. Huisman J. (2016). Similar students and different countries? An analysis of the barriers and drivers for Erasmus participation in seven countries. J. Stud. Int. Educ. 20, 184–204. doi: 10.1177/1028315315595703

[ref5] Cheung K. Yip T. L. Wan C. L. J. Tsang H. Zhang L. W. Parpala A. (2020). Differences in study workload stress and its associated factors between transfer students and freshmen entrants in an Asian higher education context. PLoS One 15:e0233022. doi: 10.1371/journal.pone.0233022, PMID: 32413088 PMC7228073

[ref6] Diseth Å. Pallesen S. Brunborg G. S. Larsen S. (2010). Academic achievement among first semester undergraduate psychology students: the role of course experience, effort, motives and learning strategies. High. Educ. 59, 335–352. doi: 10.1007/s10734-009-9251-8

[ref7] Diseth Å. Pallesen S. Hovland A. Larsen S. (2006). Course experience, approaches to learning and academic achievement. Educ. Training 48, 156–169. doi: 10.1108/00400910610651782

[ref8] Dyrbye L. N. Thomas M. R. Harper W. Massie F. S. Power D. V. Eacker A.. (2009). The learning environment and medical student burnout: a multicentre study. Med. Educ. 43, 274–282. doi: 10.1111/j.1365-2923.2008.03282.x, PMID: 19250355

[ref9] Entwistle N. (2009). Teaching for Understanding at University: Deep Approaches and Distinctive Ways of Thinking. London: Palgrave Macmillan

[ref10] Entwistle N. McCune V. Hounsell J. (2002). Approaches to studying and perceptions of university teaching-learning environments: Concepts, measures, and preliminary findings. Enhancing teaching-learning environments in undergraduate courses project. 1–19. doi: 10.13140/RG.2.2.33594.80329

[ref700] Entwistle N. J. McCune V. Hounsell J. (2003). “Investigating ways of enhancing university teaching -learning environments: Measuring students’ approaches to studying and perceptions of teaching,” in Unravelling Basic Components and Dimensions of Powerful Learning Environments. eds. De Corte E. Verschaffel L. Entwistle N. van Merrienboer J. (Oxford: Elsevier Science), 89–107.

[ref11] Entwistle N. Ramsden P. (1983). Understanding student learning. London: Croom Helm.

[ref12] Gijbels D. Van der Watering G. Dochy F. Van den Bossche P. (2005). The relationship between students’ approaches to learning and the assessment of learning outcomes. Eur. J. Psychol. Educ. 20, 327–341. doi: 10.1007/BF03173560

[ref13] Hailikari T. Virtanen V. Vesalainen M. Postareff L. (2021). Student perspectives on how different elements of constructive alignment support active learning. Active Learn. High. Educ. 146978742198916, 1–15. doi: 10.1177/1469787421989160

[ref14] Henderson M. Ryan T. Boud D. Dawson P. Phillips M. Molloy E.. (2021). The usefulness of feedback. Active Learn. High. Educ. 22, 229–243. doi: 10.1177/1469787419872393

[ref15] Hernesniemi E. Räty H. Kasanen K. Cheng X. Hong J. Kuittinen M. (2017). Burnout among Finnish and Chinese university students. Scand. J. Psychol. 58, 400–408. doi: 10.1111/sjop.12380, PMID: 28800165

[ref16] Herrmann K. J. Bager-Elsborg A. Parpala A. (2017). Measuring perceptions of the learning environment and approaches to learning: validation of the learn questionnaire. Scand. J. Educ. Res. 61, 526–539. doi: 10.1080/00313831.2016.1172497

[ref17] Hu X. Yeo G. B. (2020). Emotional exhaustion and reduced self-efficacy: the mediating role of deep and surface learning strategies. Motiv. Emot. 44, 785–795. doi: 10.1007/s11031-020-09846-2

[ref18] Jin L. Schneider J. (2019). Faculty views on international students: a survey study. J. Int. Stud. 9, 84–96. doi: 10.32674/jis.v9i1.268

[ref19] Jin L. Yang E. Zamudio G. (2021). Self-determined motivation, acculturation, academic burnout, and psychosocial well-being of Chinese international students in South Korea. Couns. Psychol. Q. 35, 1–18. doi: 10.1080/09515070.2021.1887084

[ref20] Lin S.-H. Huang Y.-C. (2012). Investigating the relationships between loneliness and learning burnout. Active Learn. High. Educ. 13, 231–243. doi: 10.1177/1469787412452983

[ref21] Lin S.-H. Huang Y.-C. (2014). Life stress and academic burnout. Active Learn. High. Educ. 15, 77–90. doi: 10.1177/1469787413514651

[ref22] Lin S.-Y. Scherz S. D. (2014). Challenges facing Asian international graduate students in the US: pedagogical considerations in higher education. J. Int. Stud. 4, 16–33. doi: 10.32674/jis.v4i1.494

[ref23] Lindblom-Ylänne S. Parpala A. Postareff L. (2019). What constitutes the surface approach to learning in the light of new empirical evidence? Stud. High. Educ. 44, 2183–2195. doi: 10.1080/03075079.2018.1482267

[ref24] Lizzio A. Wilson K. Simons R. (2002). University students’ perceptions of the learning environment and academic outcomes: implications for theory and practice. Stud. High. Educ. 27, 27–52. doi: 10.1080/03075070120099359

[ref25] Marton F. Säljö R. (1976). On qualitative differences in learning: I—outcome and process. Br. J. Educ. Psychol. 46, 4–11. doi: 10.1016/j.evalprogplan.2017.12.002

[ref26] Maslach C. Jackson S. E. Leiter M. P. (1996). Maslach Burnout Inventory Manual (3rd edn.). Menlo Park. CA: Mind Garden.

[ref27] McManus I. Keeling A. Paice E. (2004). Stress, burnout and doctors’ attitudes to work are determined by personality and learning style: a twelve-year longitudinal study of UK medical graduates. BMC Med. 2:29. doi: 10.1186/1741-7015-2-29, PMID: 15317650 PMC516448

[ref28] Meriläinen M. (2014). Factors affecting study-related burnout among Finnish university students: teaching-learning environment, achievement motivation and the meaning of life. Qual. High. Educ. 20, 309–329. doi: 10.1080/13538322.2014.978136

[ref29] Mitchell C. Del Fabbro L. Shaw J. (2017). The acculturation, language and learning experiences of international nursing students: implications for nursing education. Nurse Educ. Today 56, 16–22. doi: 10.1016/j.nedt.2017.05.019, PMID: 28623678

[ref30] OECD (2020). “What is the profile of internationally mobile students,” in Education at a Glance 2020: OECD Indicators (Paris: OECD Publishing), 226–240.

[ref31] Parpala A. Katajavuori N. Haarala-Muhonen A. Asikainen H. (2021a). How did students with different learning profiles experience ‘Normal’ and online teaching situation during COVID-19 spring? Soc. Sci. 10:337. doi: 10.3390/socsci10090337

[ref32] Parpala A. Lindblom-Ylänne S. (2012). Using a research instrument for developing quality at the university. Qual. High. Educ. 18, 313–328. doi: 10.1080/13538322.2012.733493

[ref33] Parpala A. Lindblom-Ylänne S. Komulainen E. Entwistle N. (2013). Assessing students’ experiences of teaching-learning environments and approaches to learning: validation of a questionnaire used in different countries and varying contexts. Learn. Environ. Res. 16, 201–215. doi: 10.1007/s10984-013-9128-8

[ref34] Parpala A. Lindblom-Ylänne S. Komulainen E. Litmanen T. Hirsto L. (2010). Students’ approaches to learning and their experiences of the teaching-learning environment in different disciplines. Br. J. Educ. Psychol. 80, 269–282. doi: 10.1348/000709909X476946, PMID: 19906328

[ref35] Parpala A. Mattsson M. Herrmann K. J. Bager-Elsborg A. Hailikari T. (2021b). Detecting the variability in student learning in different disciplines—a person-oriented approach. Scand. J. Educ. Res. 66, 1020–1037. doi: 10.1080/00313831.2021.1958256

[ref36] Postareff L. Mattsson M. Parpala A. (2018). The effect of perceptions of the teaching-learning environment on the variation in approaches to learning - between-student differences and within-student variation. Learn. Individ. Differ. 68, 96–107. doi: 10.1016/j.lindif.2018.10.006

[ref37] Räisänen M. Postareff L. Mattsson M. Lindblom-Ylänne S. (2020). Study-related exhaustion: first-year students’ use of self-regulation of learning and peer learning and perceived value of peer support. Active Learn. High. Educ. 21, 173–188. doi: 10.1177/1469787418798517

[ref38] Rytkönen H. Parpala A. Lindblom-Ylänne S. Virtanen V. Postareff L. (2012). Factors affecting bioscience students’ academic achievement. Instr. Sci. 40, 241–256. doi: 10.1007/s11251-011-9176-3

[ref39] Sakurai Y. (2021). Engagement in continuing subject knowledge development: a year after short-term international courses. J. Stud. Int. Educ. 26:102831532110042, 493–510. doi: 10.1177/10283153211004284

[ref40] Sakurai Y. Parpala A. Pyhältö K. Lindblom-Ylänne S. (2016). Engagement in learning: a comparison between Asian and European international university students. Compare 46, 24–47. doi: 10.1080/03057925.2013.866837

[ref41] Sakurai Y. Pyhältö K. Lindblom-Ylänne S. (2014). Are Chinese university students more likely to exhibit a surface approach to learning than other international students in Finland? J. Res. Int. Educ. 13, 135–148. doi: 10.1177/1475240914540119

[ref42] Salmela-Aro K. Kiuru N. Leskinen E. Nurmi J.-E. (2009). School burnout inventory (SBI): reliability and validity. Eur. J. Psychol. Assess. 25, 48–57. doi: 10.1027/1015-5759.25.1.48

[ref43] Salmela-Aro K. Read S. (2017). Study engagement and burnout profiles among Finnish higher education students. Burnout Res. 7, 21–28. doi: 10.1016/j.burn.2017.11.001

[ref45] Schaufeli W. B. Martínez I. M. Pinto A. M. Salanova M. Bakker A. B. (2002). Burnout and engagement in university students: a cross-National Study. J. Cross-Cult. Psychol. 33, 464–481. doi: 10.1177/0022022102033005003

[ref46] Smith S. M. Carter-Rogers K. Norris M. E. Brophy T. (2022). Students starting university: exploring factors that promote success for first-year international and domestic students. Front. Educ. 7:779756. doi: 10.3389/feduc.2022.779756

[ref47] Tackett S. Wright S. Lubin R. Li J. Pan H. (2017). International study of medical school learning environments and their relationship with student well-being and empathy. Med. Educ. 51, 280–289. doi: 10.1111/medu.13120, PMID: 27896846

[ref48] Tian M. Lu G. Li L. Yin H. (2021). International undergraduate students in Chinese higher education: an engagement typology and associated factors. Front. Psychol. 12:680392. doi: 10.3389/fpsyg.2021.680392, PMID: 34248781 PMC8260833

